# Uncovering the Roles of Clocks and Neural Transmission in the Resilience of *Drosophila* Circadian Network

**DOI:** 10.3389/fphys.2021.663339

**Published:** 2021-05-26

**Authors:** Edouard Jaumouillé, Rafael Koch, Emi Nagoshi

**Affiliations:** Department of Genetics and Evolution, Institute of Genetics and Genomics of Geneva (iGE3), University of Geneva, Geneva, Switzerland

**Keywords:** circadian rhythms, *Drosophila*, pacemaker, tetanus toxin light chain, circuit, locomotor behavior, circadian clock

## Abstract

Studies of circadian locomotor rhythms in *Drosophila melanogaster* gave evidence to the preceding theoretical predictions on circadian rhythms. The molecular oscillator in flies, as in virtually all organisms, operates using transcriptional-translational feedback loops together with intricate post-transcriptional processes. Approximately150 pacemaker neurons, each equipped with a molecular oscillator, form a circuit that functions as the central pacemaker for locomotor rhythms. Input and output pathways to and from the pacemaker circuit are dissected to the level of individual neurons. Pacemaker neurons consist of functionally diverse subclasses, including those designated as the Morning/Master (M)-oscillator essential for driving free-running locomotor rhythms in constant darkness and the Evening (E)-oscillator that drives evening activity. However, accumulating evidence challenges this dual-oscillator model for the circadian circuit organization and propose the view that multiple oscillators are coordinated through network interactions. Here we attempt to provide further evidence to the revised model of the circadian network. We demonstrate that the disruption of molecular clocks or neural output of the M-oscillator during adulthood dampens free-running behavior surprisingly slowly, whereas the disruption of both functions results in an immediate arrhythmia. Therefore, clocks and neural communication of the M-oscillator act additively to sustain rhythmic locomotor output. This phenomenon also suggests that M-oscillator can be a pacemaker or a downstream path that passively receives rhythmic inputs from another pacemaker and convey output signals. Our results support the distributed network model and highlight the remarkable resilience of the *Drosophila* circadian pacemaker circuit, which can alter its topology to maintain locomotor rhythms.

## Introduction

**FIGURE 1:**
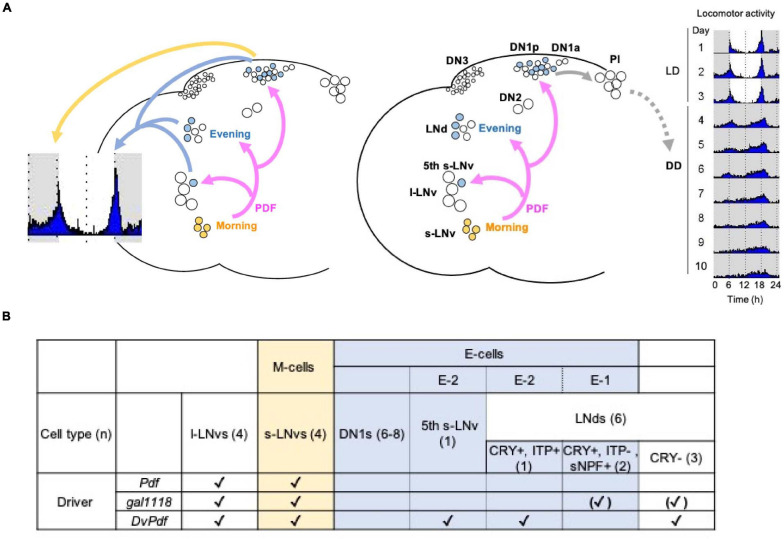
Current model of the circadian pacemaker circuit organization in Drosophila melanogaster. **(A)** Four PDF-positive s-LNvs constitute the Morning (M)-cells and the Evening (E)-cells consist of the 5th s-LNv, three CRY-positive LNds and 6–8 DN1ps. Left, in LD and constant temperature, the M-cells control the morning activity peak through PDF signaling onto the DN1ps. The E-cells drive the evening activity peak. In certain environmental conditions, the DN1ps are able to drive both morning and evening peaks. Right, in DD and constant temperature, the M-cells determine the pace of the locomotor rhythms via PDF signaling to the E-cells. However, the coupling between the M- and E-cells are within a limited temporal range. Among the E-cells, CRY- and sNPF-positive, ITP-negative 2 LNds [E-1, see **(B)**] are strongly coupled to the oscillation of the M-cells, whereas the 5th s-LNv and one ITP-positive LNd (E-2) are weakly coupled. One of the locomotor output circuits is found downstream of the DN1ps, which are connected to neuroendocrine cells in the pars intercerebralis (PI). **(B)** The identity of the M- and E-cells and the expression patterns of the GAL4 drivers used in this study. *Pdf-GAL4* is expressed in the l- and s- LNvs (Renn et al., 1999). *gal1118* is expressed in the l- and s-LNvs and weakly in the LNds. However, expression in the LNds is detectable only in the flies homozygous for *gal1118* (Blanchardon et al., 2001). *DvPdf-GAL4* is expressed in all the LNvs and one CRY-positive, ITP-positive LNd and three CRY-negative LNds (Bahn et al., 2009; Schubert et al., 2018). (n) indicates the number of cells per hemisphere.

Circadian oscillators across the evolutionary tree operate using transcriptional-translational feedback loops (Hurley et al., 2016). In *Drosophila*, the transcriptional activators CLOCK/CYCLE (CLK/CYC) drive the expression of the *period* (*per*) and timeless (*tim*) genes. The PER-containing complexes inhibit the activity of CLK/CYC, thereby forming a principal negative feedback loop. Furthermore, positive- and negative- feedback loops created by PAR DOMAIN PROTEIN 1 (PDP-1) and VRILLE (VRI) on *Clk* expression are, respectively, coupled with the main negative-feedback loop to ensure the generation of 24 h rhythms (Hardin, 2011). Circadian pacemaker neurons are classified into anatomically and functionally diverse subclasses: small and large lateral ventral neurons (s- and l-LNvs), lateral dorsal neurons (LNds), lateral posterior neurons (LPNs) and three groups of dorsal neurons (DN1s, DN2s, DN3s) (Helfrich-Forster et al., 2007). The s-LNvs are further divided into four neurons that express the neuropeptide pigment dispersing factor (PDF) and one PDF-negative neuron (5th s-LNv) (Figure 1A).

Previously many studies have posited that the PDF-positive s-LNvs as the Morning-oscillator (M-oscillator; M-cells) that anticipates dawn (Helfrich-Forster, 1998; Grima et al., 2004; Stoleru et al., 2004, 2005). Additionally, these classical studies showed that the M-cells are required for the generation and setting the pace of free-running locomotor rhythms in constant darkness (DD). A separate group of pacemaker neurons named Evening (E)-oscillator (E-cells), including the PDF-negative 5th s-LNv, LNds and some of the DN1s (DN1s), controls evening bout of activity. PDF released from the M-cells functionally couples M- and E-oscillators to generate coherent behavioral output (Grima et al., 2004; Stoleru et al., 2004; Picot et al., 2007). Therefore, loss of PDF or PDF receptor (PDFR) results in the absence of the morning anticipation, advancing the evening peak, short period locomotor rhythms with very weak rhythmicity (Renn et al., 1999; Hyun et al., 2005; Lear et al., 2005; Shafer and Taghert, 2009; Yoshii et al., 2009; Choi et al., 2012).

However, this somewhat simplistic view on the circadian network organization and the dominant role of the PDF-positive s-LNvs has been challenged by accumulating evidence. Works that characterized the property of the E-cells in response to light have redefined the 5th s-LNv, three Cryptochrome (CRY)-positive LNds and 6–8 posterior subgroup of DN1s (DN1ps) as the E-cells (Rieger et al., 2006; Picot et al., 2007; Figure 1A). More precisely, since the clocks restricted only in the CRY-positive, PDF-negative 4 Lateral Neurons, i.e., the 5th s-LNvs and 3 CRY-positive LNds, are able to drive evening activity peak, they were defined as the Lateral Neuron-Evening oscillator (LN-EO) (Picot et al., 2007). Morning anticipation requires the presence of the M-cells (Renn et al., 1999; Stoleru et al., 2004) and *per* expression only in the M-cells is sufficient to restore morning anticipation in *per* null (*per*^0^) mutants (Cusumano et al., 2009) at least under the 12 h:12 h LD cycle and constant temperature of around 25∘C (Menegazzi et al., 2020). However, *per*^0^ flies expressing *per* in all neurons except the LNvs exhibit morning anticipation (Stoleru et al., 2004). Genetic rescue of *per* in *per*^0^ flies with the *Mai179-GAL4* driver, which is expressed in the s- and l-LNvs, the 5th LNv and 3 CRY-positive LNds (Cusumano et al., 2009) or with the *DvPdf-GAL4* driver expressed in the s- and l-LNvs, the 5th LNv, three CRY-negative LNd and one LNd co-expressing CRY and the ion transport peptide (ITP) (Figure 1B; Guo et al., 2014) restores morning anticipation. However, *per* rescue only in the LN-EO with the combination of *Pdf-GAL80* and *Mai179-GAL4* does not restore morning anticipation (Cusumano et al., 2009).

The puzzle that *per* rhythms in the M-cells are not necessary but clocks in the LN-EO are not sufficient for driving morning activity peak was solved in the studies focusing on the role of DN1ps (Zhang L.Y. et al., 2010; Zhang Y. et al., 2010). Strikingly, *per* expression in the DN1ps alone is sufficient to restore morning peak in *per*^0^ flies (Zhang Y. et al., 2010). The ability of DN1ps to drive morning activity depends on the PDF signaling (Zhang L.Y. et al., 2010). Furthermore, under dim light conditions, *per* expression in the DN1ps alone can also drive evening anticipatory activity (Zhang Y. et al., 2010). Taken together, these findings have shown that DN1ps are the major output route of the M-cells and can also serve as M- and E- oscillators, depending on the environmental conditions (Lamaze and Stanewsky, 2019).

Manipulating the speed of the M-cells’ clocks changes the pace of subsets of the E-cells and free-running period in DD, within a limited temporal range (Guo et al., 2014; Yao and Shafer, 2014). The pace of DN1ps is strongly controlled by the speed of clocks in the M-cells in DD (Chatterjee et al., 2018). Among the LN-EO, the pace of two LNds co-expressing CRY and the short neuropeptide F (sNPF) (E-1 group, Figure 1B) is controlled by the clocks in the M-cells, whereas the 5th LNv and one LNd co-expressing CRY and the ITP ITP do not follow M-cell’s rhythms (E-2 group, Figure 1B; Yao and Shafer, 2014; Chatterjee et al., 2018). When the discrepancy of the periods between the M-cells and PDF-negative clock neurons is larger than ∼ 2.5 h, the M-cells no longer dictates the coherent behavioral rhythms (Yao and Shafer, 2014). Moreover, electrical silencing or disruption of clocks of non-LNv pacemaker neurons deteriorates locomotor rhythms without affecting clockwork in the M-cells (Bulthuis et al., 2019). Behavioral period in DD can be also modified by manipulating the pace of non-M cells (Dissel et al., 2014). CRISPR knockout of *per* or *tim* in the M-cells do not affect free-running rhythms, whereas ablation of *per* or *tim* in both M and E-cells render flies arrhythmic (Delventhal et al., 2019; Schlichting et al., 2019). Collectively, these works have demonstrated that behavioral period and rhythmicity are determined by the action of multiple independent oscillators coordinated by network interaction, rather than by a single dominant oscillator.

In this paper, we attempt to provide further evidence to the revised model of the circadian circuit organization. To this end, we exclusively use conditional approaches to disrupt molecular clocks or neural communication in adulthood, in order to distinguish the outcome caused by the effects during adulthood from any process during development. We find that disruption of molecular clocks in the M-oscillator or both M-oscillator and part of the LN-EO only gradually weakens locomotor rhythmicity in DD, which contrasts the immediate loss of morning activity peak. Suppressing neuronal output of the M-cells in adulthood reduces the power of the locomotor rhythmicity in DD also gradually. However, disruption of both molecular clockwork and neural output of the M-oscillator leads to an immediate arrhythmia. These results indicate that the M-oscillator can be a master pacemaker or an output pathway of other pacemakers, thus largely support the emerging consensus that circadian circuit is composed of multiple oscillators that can flexibly change the network topology.

## Materials and Methods

### Fly Strains

*Drosophila* were reared at 25°C on a corn-meal medium under 12 h:12 h light-dark (LD) cycles. *UAS-CLK*Δ (Tanoue et al., 2004) was a gift from Jadwiga Giebultowicz. *UAS-TNT* (*UAS-TNT-G*) (Kaneko et al., 2000) was a gift from Jeff Hall. UAS-*per RNAi* (*perCt-IR*) (Martinek and Young, 2000), *Pdf-GAL4* (Park et al., 2000), *gal118* (Blanchardon et al., 2001), *DvPdf-Gal4* (Bahn et al., 2009), and *tubulin-GAL80ts* (McGuire et al., 2004) were described previously.

### Behavioral Assays

The locomotor behavior assay was performed as described in Beuchle et al. (2012) using the Drosophila Activity Monitoring (DAM) System (Trikinetics, Waltham, MA), except that assays were performed at 29∘C in the experiments with adult-restricted conditional GAL4 induction and at 18∘C for developmental GAL4 induction. For adult-restricted GAL4 induction, flies were crossed and raised at 18∘C until 2 days after eclosion. Male flies of appropriate genotypes were then collected and placed in the DAM monitors and assayed for locomotor activity at 29∘C. Flies were first entrained in 12 h:12 h-LD cycles for 4 days and then released in DD for 10–12 days. The light intensity of the incubator was approximately 1000 lux. For experiments with developmental GAL4 expression, flies were raised at 29∘C until 2 days after eclosion, and then the collected flies were assayed at 18∘C. In both sets of experiments, behavioral data were analyzed from the second day in LD. Two to four independent experiments were performed for each genotype. The numbers of flies used in the behavioral assays are indicated in Table 1. The behavioral data were analyzed using FaasX software (Blanchardon et al., 2001). The flies with power over 20 and width over 2.5 h according to the χ^2^ periodogram analysis were defined as rhythmic. The significance threshold was set to 5%. Morning anticipation index (M-index) was calculated as described in Im and Taghert (2010) with minor modifications. The M-index was calculated for individual flies as (sum of activity over 3 h before lights on)/(sum activity over 6 h before lights on) at each day from LD2 to LD4, and the 3 values were averaged to obtain the mean M-index of an individual fly. The mean M-indices were pooled per genotype and presented as boxplots.

**TABLE 1:** Free-running locomotor rhythms in flies with adult-restricted genetic manipulations.

**Genotype**	**n**	**DD1-5**			**DD6-10**		
		
		**Period ± SEM (h)**	**Power ± SEM**	**%R**	**Period ± SEM (h)**	**Power ± SEM**	**%R**
*Pdf-GAL4/*+; *tub-GAL80ts/*+	124	23.9 ± 0.3	95.2 ± 4.3	95.2	24.3 ± 0.1	79.1 ± 2.7	88.1
*gal1118/tub-GAL80ts*	60	23.4 ± 0.2	89.4 ± 4.3	96.6	23.4 ± 0.3	46.6 ± 4.3	72.7
*UAS-Clk*Δ/+; *tub-GAL80ts/*+	56	23.4 ± 0.2	105.2 ± 13.1	91.1	23.5 ± 0.3	61.95 ± 9.3	55.0
*DvPdf-GAL4/*+; *tub-GAL80ts*	25	23.6 ± 0.1	66.3 ± 8.4	76.0	24.1 ± 0.8	35.6 ± 3.7	52.6
*UAS-Clk*Δ*/Pdf-GAL4; tub-GAL80ts/* +	59	24.4 ± 0.8	74.9 ± 2.31	98.3	24.0 ± 1.1	37.7 ± 2.0	49.1
*UAS-Clk*Δ/+; *gal1118/tub-GAL80ts*	63	23.9 ± 0.3	74.25 ± 2.8	95.2	23.3 ± 0.2	52.6 ± 7.9	25.4
*UAS-Clk*Δ*/DvPdf-GAL4; tub-GAL80ts/* +	26	24.0 ± 0.5	72.2 ± 12.9	76.9	25.1 ± 0.8	34.6 ± 13.9	16.7
*UAS-per RNAi/Pdf-GAL4; tub-GAL80ts/* +	59	23.4 ± 0.1	97.6 ± 15.8	98.3	23.6 ± 0.1	73.2 ± 14.3	75.4
*UAS-TNT/*+; *tub-GAL80ts/*+	59	23.5 ± 0.0	84.7 ± 1.3	88.1	23.5 ± 0.1	61.0 ± 5.4	63.2
*UAS-TNT/*+; *gal1118/tub-GAL80ts*	29	23.6 ± 0.1	50.1 ± 0.0	75.9	22.5 ± 0.0	11.1 ± 3.6	12.5
*UAS-Clk*Δ*/UAS-TNT; gal1118/tub-GAL80ts*	40	23.4 ± 0.1	18.7 ± 9.0	3.1	–	2.98 ± 1.2	0

*n, number of flies; %R, % of rhythmic flies.*

### Immunohistochemistry, Microscopy, and Image Analysis

Anti-PER and PDF immunostaining of fly brains was performed as described previously (Shafer et al., 2002). The brains were imaged using a Leica SP5 confocal microscope and images were analyzed using Fiji/Image J software (National Institutes of Health). To quantify PER staining intensity, sum slices projections were generated from 2 μm z-section confocal images, and the mean pixel value of each cell and background pixel value was measured. The mean pixel value of each cell in a given subgroup was calculated by subtracting the mean pixel value of the background and plotted as relative intensity normalized to the value of the control group at CT0. PDF levels in the s-LNv dorsal terminals were measured as described in Kozlov et al. (2017). Briefly, the region of interest (ROI) for the axonal termini (from the tip of the s-LNv dorsal termini until where the terminal arbors first branch) was specified manually with the polygon selection tool in Fiji and the intensity sum within each ROI was measured. The representative confocal images were maximum projections generated from the same confocal z-series used for the quantification.

### Statistical Analysis

Statistical analysis and data visualization were performed using GraphPad Prism (9.0). A *p*-value < 0.05 is considered a statistically significant test result. Asterisks indicate *p*-values, where ^∗^*p* < 0.05, ^∗∗^*p* < 0.01, ^∗∗∗^*p* < 0.001, and ^****^p < 0.0001. ns indicates a non-significant test result. Data were first tested for normality with D’Agostino-Pearson K2 test, and normally distributed data sets were analyzed using parametric tests (ANOVA and unpaired *t*-test with Welch’s correction) and non-normally distributed data were analyzed with non-parametric tests (the Kruskal-Wallis test and Mann-Whitney *U*-test).

Welch’s *t*-test and Mann-Whitney *U*-test were used for pairwise comparisons of the behavioral rhythmicity between control and test genotype groups, depending on the distribution of each data set. Morning anticipation indices were compared using the Kruskal-Wallis test with Dunn’s multiple comparisons test. Signal intensities of immunofluorescence images were compared using the multiple unpaired *t*-test with Welch’s correction or ANOVA with Sidak’s multiple comparison’s test.

## Results

### Disruption of Molecular Clocks in Adult Morning/Master-Oscillator Has a Modest Effect on Free-Running Locomotor Rhythms

**FIGURE 2:**
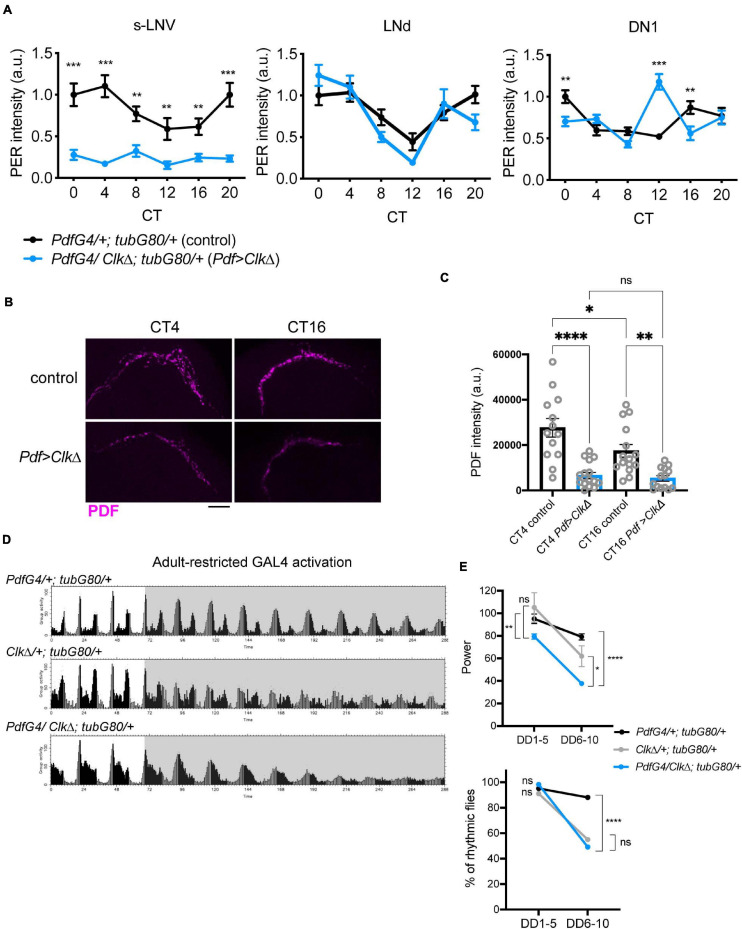
Blocking M-oscillator clocks by conditional expression of CLKΔ has little effects on free-running locomotor rhythms. *UAS-Clk*Δ was expressed in the LNvs only during adulthood with *Pdf-GAL4*, *tub-GAL80^*ts*^* and a temperature shift from 18to 29°C. **(A)** The brains were stained for PER and PDF on DD3 and the levels of PER in the s-LNvs, DN1s and LNds were quantified. *n* = 20–27 hemispheres per group. **p < 0.01, ***p < 0.001 by multiple unpaired *t*-test with Welch’s correction, comparing two genotypes at each timepoint. **(B,C)** PDF levels in the s-LNv dorsal terminals at two timepoints on DD3. Adult-restricted *Clk*Δ in the LNvs significantly reduced PDF levels and disrupted its rhythm. Scale bar, 20 μm. *n* = 12–15 per group. *p < 0.05, **p < 0.01, and ***p < 0.00 by 2-way ANOVA with Sidak’s multiple comparison test. **(D)** Group average locomotor activities of the flies in LD (white background) and DD (gray background). **(E)** The power of rhythmicity and the percentage of rhythmic flies during the first 5 days in DD (DD1-5) and from the 6th to 10th days in DD (DD6-10) in the flies expressing *Clk*Δ (*PdfG4/Clk*Δ; *tubG80/* + stands for *Pdf-GAL4/UAS- Clk*Δ; *tub-GAL80^*ts*^*/ +) and in two control groups (*PdfG4/*+; *tubG80/*+ indicates *Pdf-GAL4/*+; *tub-GAL80^*ts*^/*+ and *Clk*Δ/+; *tubG80/* + indicates UAS-*Clk*Δ; *tub-GAL80^*ts*^*/+). The difference in the rhythm power was tested using the unpaired *t*-test with Welch’s correction and Mann-Whitney *U*-test *p < 0.05, **p < 0.01, and ****p < 0.0001. The percentages of rhythmic flies were compared using Fisher’s exact test. ****p < 0.0001. ns, not significant.

To conditionally eliminate molecular clockwork only during adulthood in the LNvs, we drove the expression of *CLK*Δ, a dominant-negative mutant of CLK (Tanoue et al., 2004), using the combination of *Pdf-GAL4* and temperature sensitive GAL80 expressed ubiquitously under the *tubulin* promoter (*tub-GAL80^*ts*^*). The flies were raised at 18∘C until 2 days after eclosion and adult male flies were maintained at 29∘C during the subsequent experiments (McGuire et al., 2004). To verify if this manipulation effectively blocks molecular clockwork, the brains of flies were immunolabeled with anti-PER and anti-PDF antibodies every 4 h on the third day in constant darkness (DD3) following an entrainment to 12 h:12 h light-dark (LD) cycles for 4 days (Supplementary Figure 1). As expected, PER levels in the s-LNvs were significantly reduced and arrhythmic on DD3. In DN1s, PER levels peaked at CT12 but did not show 24 h rhythmicity. This observation is congruent with the notion that molecular rhythms in the s-LNvs affect rhythmicity of the DN1s (Nitabach et al., 2006; Zhang L.Y. et al., 2010; Beuchle et al., 2012; Jaumouille et al., 2015). PER levels and oscillations in the LNds were not different between control and CLKΔ-expressing flies (Figure 2A). Moreover, PDF signal at the s-LNv dorsal termini was reduced and arrhythmic in CLKΔ-expressing flies (Figures 2B,C). This observation confirms the previous finding that *Clk* controls PDF levels and accumulation rhythms via regulating *vri* expression in adult s-LNvs (Gunawardhana and Hardin, 2017).

Having validated that adult-restricted expression of CLKΔ in the LNvs effectively abolishes molecular clockwork, we next assayed locomotor behavior of these flies. Unexpectedly, CLKΔ driven with *Pdf-GAL4* and *tub-GAL80ts* had a modest effect on free-running locomotor rhythms at least up to DD10. Most (98.3%) of the *Pdf-GAL4/CLK*Δ; *tub-GAL80ts/*+ flies were rhythmic until DD5, despite with a reduced rhythm power. Their rhythms damped significantly after DD6 compared to control flies (Figures 2D,E and Table 1). To verify the effect of adult-restricted CLKΔ expression in the LNvs, we next turned to the *gal1118* driver (Malpel et al., 2004). *Gal1118* is expressed in the s- and l-LNvs and weakly expressed in 5 LNds, but the expression in the LNds is detectable only in the flies homozygous for *gal1118* (Malpel et al., 2004; Figure 1B). Therefore, we used one copy of *gal1118* in combination with *tub-GAL80ts* to express CLKΔ only during adulthood. These flies remained highly rhythmic during the first 5 days in DD. After DD6, a high proportion of these flies became arrhythmic (Table 1 and Supplementary Figures 2A,B). These results indicate that behavioral rhythms in DD only gradually dampen over several days when clocks in the LNvs are disrupted in adulthood.

**FIGURE 3:**
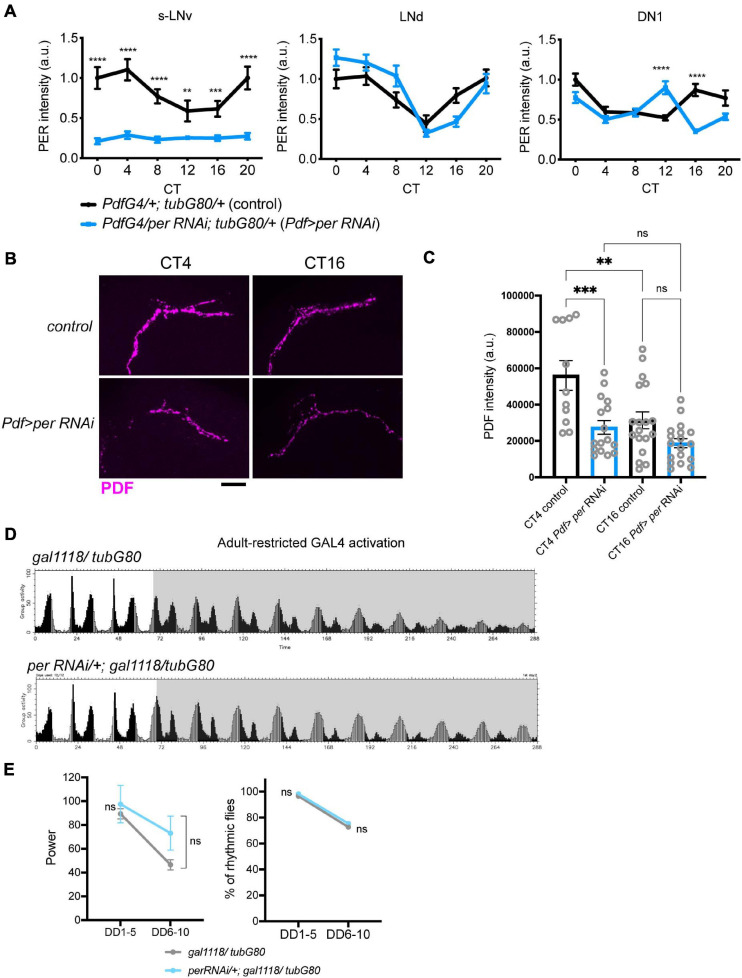
PER knockdown in adult M-oscillators disrupts molecular clocks but has little effects on free-running locomotor rhythms. **(A)** UAS-*per RNAi* was expressed during adulthood with a combination of *Pdf-GAL4*, *tub-GAL80^*ts*^*. PER levels were quantified on DD3 in the s-LNvs, DN1s and LNds. *n* = 14–19 per group. **p < 0.01, ****p < 0.0001 comparing two genotypes at each timepoint by multiple unpaired *t*-test with Welch’s correction. **(B,C)** PDF levels in the s-LNv dorsal terminals at two timepoints on DD3. Representative confocal images **(B)** and quantification **(C)** of indicated genotypes. Scale bar, 20 μm. *n* = 11–18 hemispheres per group. **p < 0.01, ***p < 0.001 by 2-way ANOVA with Sidak’s multiple comparison test. **(D)** Group average locomotor activities of the flies with adult-restricted expression of *per* RNAi driven with the combination of *gal1118* and *tub-GAL80^*ts*^* and the control group carrying only the driver in LD and DD. **(E)** Left, power of rhythmicity in DD1-5 and DD6-10 in the indicated genotypes. No significant differences were found between two groups by the unpaired *t*-test with Welch’s correction. Right, percentage of rhythmic flies in DD1-5 and DD6-10. No significant differences were found by Fisher’s exact test.

To corroborate these findings, we also used *UAS-RNAi* against PER as an alternative method to temporarily block molecular clockwork. *UAS-PER RNAi* was driven in the LNvs with *Pdf-GAL4* or *gal1118* and *tub-GAL80ts* and its expression was induced by the temperature shift from 18 to 29∘C 2 days after eclosion. For behavioral assays we used *ga1118*, because many flies carrying *Pdf-GAL4*, *tub-GAL80ts*, and *UAS-PER RNAi* did not survive until the end of behavioral recording for unknown reasons. As with conditional CLKΔ expression, this treatment eliminated PER expression and rhythms in the s-LNvs, verified by anti-PER and anti-PDF immunostaining on DD3 (Supplementary Figure 1 and Figure 3A). Levels and rhythms of PDF accumulation at the dorsal termini of the s-LNvs were also reduced (Figures 3B,C). This observation is congruent with a previous report that CLK represses *pdf* transcription (Mezan et al., 2016) because PER knockdown should increase CLK/CYC-transcriptional activity (Yu and Hardin, 2006). Free-running locomotor rhythmicity of these flies were not disrupted compared to controls at least up to DD10 (Figures 3D,E and Table 1), consistent with the results of adult-restricted, LNv-targeted CLKΔ expression (Figures 2D,E, Table 1, and Supplementary Figures 2A,B).

**FIGURE 4:**
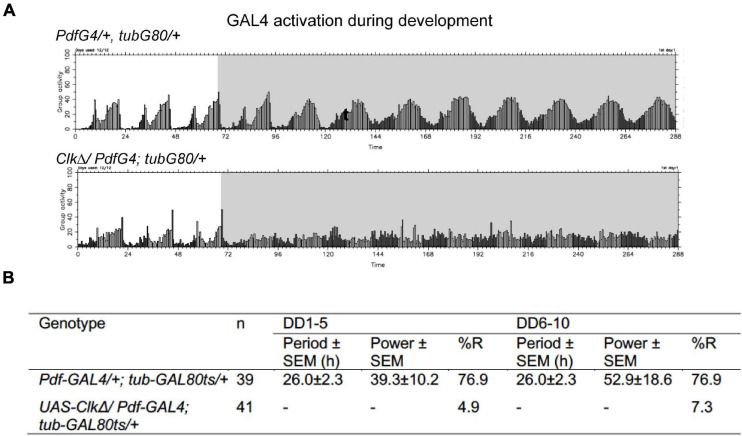
CLKΔ expression during development in the M-cells irreversibly disrupts locomotor rhythms in adulthood. **(A,B)** *UAS-Clk*Δ was expressed with *Pdf-GAL4*, *tub-GAL80^*ts*^* only during development. **(A)** Group average locomotor rhythms assayed at 18∘C following the developmental CLKΔ expression. **(B)** Percentage of rhythmic flies in DD1-5 and DD6-10 in the flies following developmental CLKΔ expression and controls.

The presence of functional clocks in developing LNvs is required for driving normal locomotor rhythms in adulthood (Goda et al., 2011; Beuchle et al., 2012). Consistent with this notion, conditional expression of CLKΔ only during development until eclosion in the LNvs resulted in an immediate behavioral arrhythmia in DD (Figures 4A,B). This finding verifies that conditional expression of CLKΔ is an effective tool to disrupt molecular clocks and highlights the differential requirements for LNvs’ clocks during development and adulthood in the functioning of the pacemaker circuit. Previous studies have shown that inactivating *per* in pacemaker neurons during development does not affect behavioral rhythms of adult flies but depleting CYC during development abolishes locomotor rhythms in adults (Ewer et al., 1990; Goda et al., 2011). Our results are in agreement with these observations and support that CLK/CYC activity has non-clock roles during development.

**FIGURE 5:**
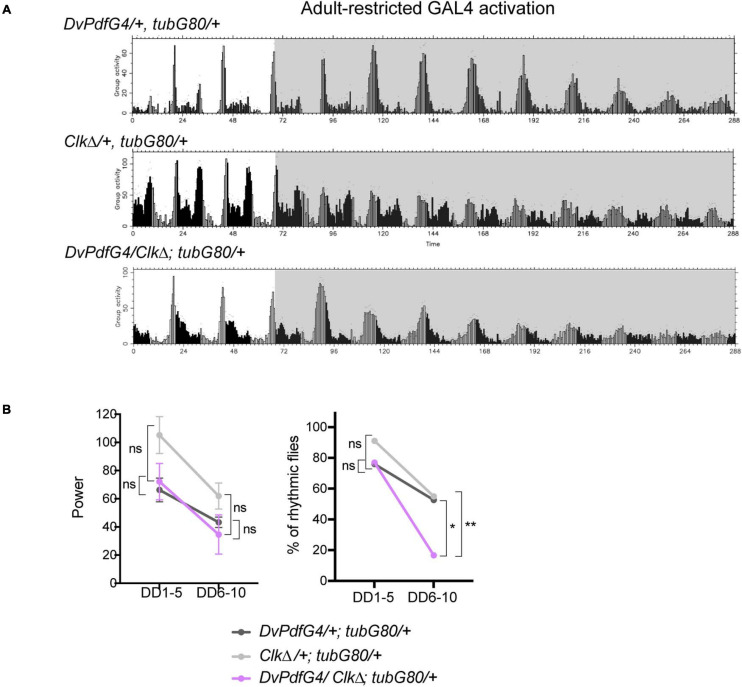
Adult-restricted expression of CLKΔ with the *DvPdf-GAL4* driver has little effects on free-running locomotor rhythms. **(A)** Group average locomotor activity in LD and DD in the flies expressing CLKΔ with the *DvPdf-GAL4* driver during adulthood and their controls. **(B)** Left, the rhythm power of flies in DD1-5 and DD6-10. Right, percentage of rhythmic flies. *p < 0.05, and **p < 0.01 by Fisher’s exact test.

How could locomotor rhythms persist several days under constant conditions while the M-cells are molecularly arrhythmic? As proposed by a number of other studies (Guo et al., 2014; Yao and Shafer, 2014; Bulthuis et al., 2019; Delventhal et al., 2019; Schlichting et al., 2019), we hypothesized that other pacemaker neurons compensate arrhythmic LNvs to drive free-running rhythms. In particular, recent studies have described that CRISPR-mediated ablation of molecular clocks in the LNvs does not cause strong behavioral phenotypes in DD, whereas clock knockout in the M- and all or part of LN-EO cells using *Mai179-GAL4* or *DvPdf-GAL4* significantly reduces behavioral rhythmicity (although the data of the *DvPdf-GAL4*-mediated clock knockout are not displayed) (Delventhal et al., 2019; Schlichting et al., 2019). Therefore, LN-EO cells are the likely candidates of the surrogate main pacemaker. To test this idea, we next expressed CLKΔ with the *DvPdf-GAL4* driver during adulthood. The majority of these flies remained rhythmic until DD5, thereafter became arrhythmic (Figures 5A,B and Table 1). However, the average rhythm power of *DvPdf-GAL4/CLK*Δ; *tub-GAL80ts/*+ flies were approximately the same as that of two control groups until DD10. Since *DvPdf-GAL4* is expressed in the M-cells and two out of four LN-EO cells (Figure 1B), these observations suggest the possibility that clocks in PDF-negative pacemaker neurons, including two LNds that do not express *DvPdf-GAL4* (i.e., CRY-positive, sNPF-positive LNds), compensate the loss of clocks in the M-cells and maintain rhythmic locomotor output for several days.

### Neural Output and Clocks of the M-Oscillator Are Additively Required to Maintain Robust Locomotor Rhythms

**FIGURE 6:**
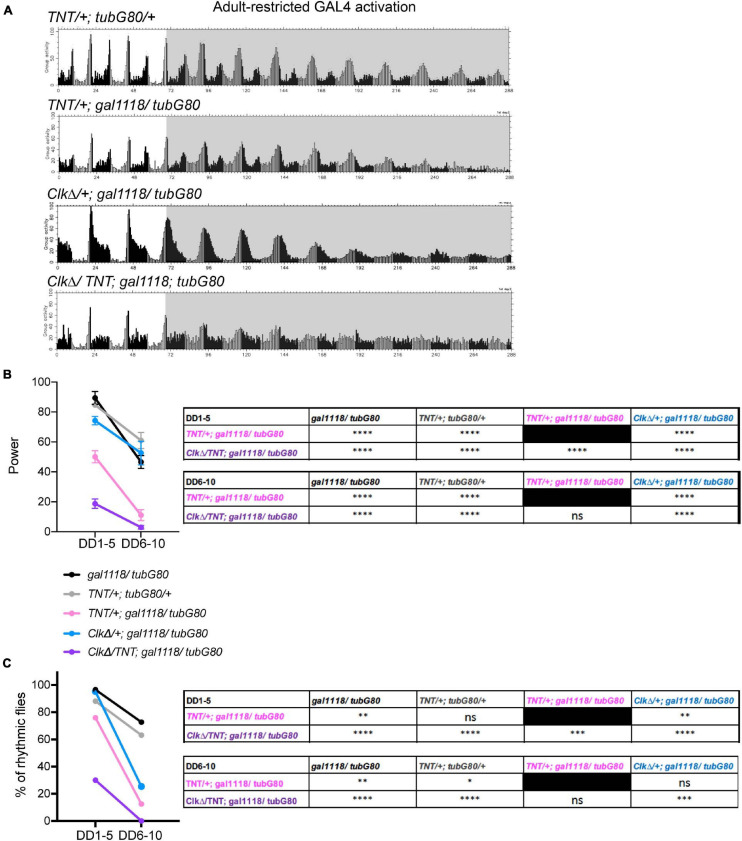
Intact neuronal output of the M-oscillator is required for sustaining robust free-running locomotor rhythms. **(A)** Group average locomotor activity of the flies expressing TNT, CLKΔ or TNT and CLKΔ in the LNvs with the combination of *gal1118* and *tub-GAL80^*ts*^* during adulthood and a control group (*UAS-TNT/* + ; *tub-GAL80^*ts*^/* +). The activity of another control group, *gal1118*/*tub-GAL80^*ts*^*, is displayed in Figure 3D. **(B)** Power of rhythmicity of the flies of indicated genotypes in DD1-5 and DD6-10. The unpaired *t*-test with Welch’s correction and Mann-Whitney *U*-test were used to compare two genotypes at the same time point. The tables show the statistical test results, in which groups labeled in the row are compared with those in the column. ^*⁣*⁣**^p < 0.0001. ns, not significant. **(C)** The percentage of rhythmic flies in DD1-5 and DD6-10. The genotypes are as in **(B)**. Results of Fisher’s exact test for pairwise comparisons are shown in the table. ^∗^*p* < 0.05, ^∗∗^*p* < 0.01, ^∗∗∗^*p* < 0.001, and ^*⁣*⁣**^p < 0.0001. ns, not significant.

Our results thus far are in line with previous works and suggest that non-M-cells can output behavioral rhythms independently of the M-cells, or they input signals to the M-cells, which then output behavioral rhythms without the need of molecular clocks in the M-cells. The possibility that non-M-cells output behavioral rhythms via M-cells is backed by the evidence that presence of the s-LNvs is essential for locomotor rhythms (Helfrich-Forster, 1998; Stoleru et al., 2004). To test this hypothesis further, we sought to block neuronal output of the M-cells with or without disrupting molecular clocks in the M-cells. We first blocked output of the LNvs during adulthood by conditionally expressing tetanus toxin light chain (TNT) (Sweeney et al., 1995) using the combination of *gal1118* and *tub-GAL80^*ts*^*. *Gal1118* was used instead of *Pdf-GAL4* due to the ease in establishing the desired genotypes in co-expression experiments. TNT is a protease that cleaves n-synaptobrevin, syntaxin or SNAP-25, thereby inhibits synaptic transmission (Sweeney et al., 1995) and neuropeptide release (Ding et al., 2019). These flies exhibited locomotor rhythms with a significantly reduced power already during the first 5 days in DD. The power of the rhythmicity was further reduced after DD6 (Figures 6A,B). Approximately 25% of these flies were arrhythmic before DD5, and thereafter approximately 80% of them became arrhythmic (Figure 6C and Table 1). This finding is largely congruent with the results presented in Kaneko et al. (2000), where the same *UAS-TNT* (*UAS-TNT-G*) line used in the present study was constitutively driven with *Pdf-GAL4*. In contrast, one study reported that constitutive expression of TNT with *Pdf-GAL4* had no effect on both LD and DD behavior, using another *UAS-TNT* insert, *UAS-TNT-E* (Umezaki et al., 2011). This discrepancy is likely attributed to the difference in expression levels, as *UAS-TNT-G* renders a higher level of expression than *UAS-TNT-E* (Kaneko et al., 1997).

We next expressed both CLKΔ and TNT in the LNvs using *gal1118* during adulthood. Strikingly, these flies became immediately arrhythmic in DD (Figures 6A–C and Table 1). In other words, loss of neural transmission and loss of molecular clockwork in adult LNvs cumulatively cause the rapid decline of rhythmic behavioral output. This finding further suggests that molecular clocks and neural output function of the M-cells are independent components that add up to enable robust rhythmic behavioral output in DD. Even when clocks are disrupted, the M-cells can receive inputs from other pacemakers and transmit output signals to the output circuit. When TNT is expressed in the M-cells, two scenarios are possible: a surrogate master pacemaker bypasses the M-cells to control locomotor output; or the M-cells still produce output signals via TNT-insensitive transmitters/peptides or by electrical coupling via gap junctions (Schneider and Stengl, 2006; Ramakrishnan and Sheeba, 2020).

### Morning Anticipation Is Independent of PER Rhythms but Requires CLK in the M-Oscillator in Adulthood

**FIGURE 7:**
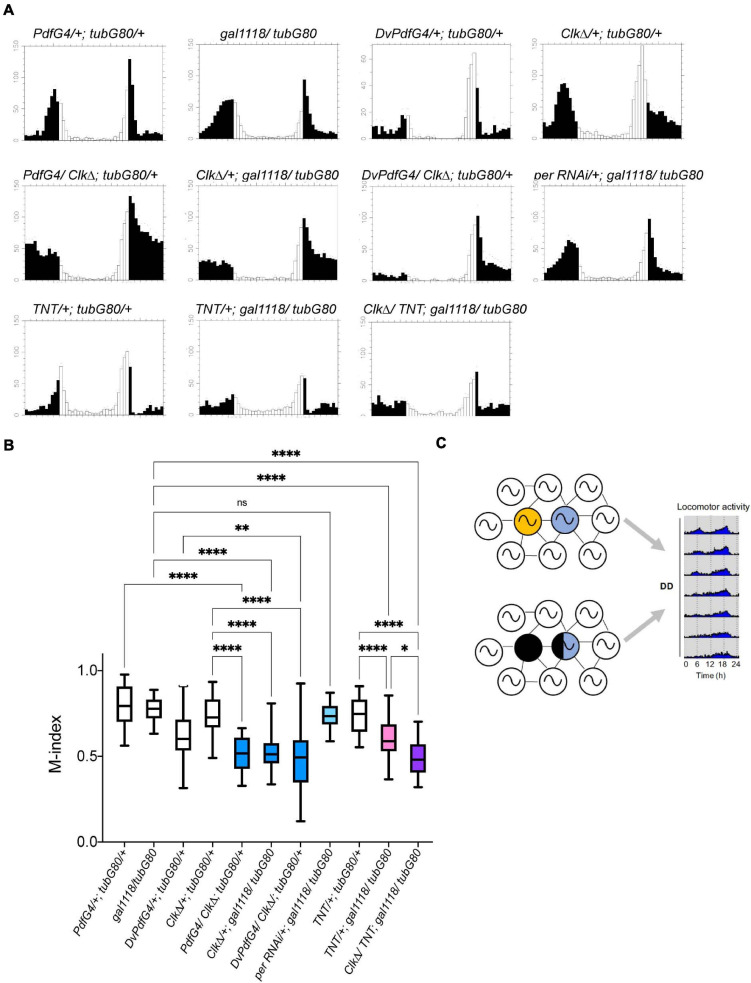
LD-entrained activities of flies with the adult-restricted disruption of clocks or neural output of the M-cells. **(A)** Average activities of flies of the indicated genotypes in LD. White bars indicate activity levels during the light period and black bars represent the dark period. **(B)** M-indices of the flies of indicated genotypes. Whiskers indicate 5th and 95th percentile and the line inside the box indicates the median. ^∗^*p* < 0.05, ^∗∗^*p* < 0.01, ^∗∗∗^*p* < 0.001, and ^*⁣*⁣**^p < 0.0001 by the Kruskal-Wallis test with Dunn’s multiple comparisons test. **(C)** A schematic summarizing the finding of this study. Circadian pacemaker network consists of diverse classes of clock-containing pacemaker neurons, including the M-oscillator (yellow) and the E-oscillator (blue). The traditional model postulates that the M-cells (yellow) drive free-running locomotor rhythms via synchronizing the pace of the E-cells. In this study, we show that locomotor rhythms can free-run several cycles while clocks in the M-cells and part of the E-cells are disrupted during adulthood, supporting the emerging view that the circadian circuit can flexibly assign different neuronal subgroups the pacemaking role to maintain rhythmic locomotor output.

It has been shown that morning anticipatory activity requires the presence of the M-cells and PDF neuropeptide (Renn et al., 1999; Grima et al., 2004; Shafer and Taghert, 2009) but can be observed in the absence of *per* rhythms within the M-cells (Stoleru et al., 2004). We examined whether morning anticipation is impaired when molecular clocks are disrupted during adulthood in the LNvs. Adult-restricted PER RNAi in the LNvs, which abolishes PER rhythms and reduces PDF levels and rhythms in the s-LNv axonal termini (Figures 3A–C), did not impair morning anticipation (Figures 7A,B, compare *perRNAi/*+; *gal1118/tubG80* and *gal1118/tubG80*). These results are congruent with the report that *per* expression in the M-cells is not required for morning anticipation (Stoleru et al., 2004). However, the morning anticipation index (M-index) (Sheeba et al., 2010) was significantly reduced when CLKΔ was conditionally driven in adulthood with *Pdf-GAL4* or *gal1118* (Figures 7A,B, see *PdfG4/Clk*Δ; *tubG80/*+, *Clk*Δ/+; *gal1118/tubG80, PdfG4/*+; *tubG80/*+, *gal1118/tubG80*, and *Clk*Δ/+; *tubG80/*+). Similarly, adult-restricted CLKΔ expression using *Dvpdf-GAL4* reduced morning anticipation compared with controls (Figures 7A,B, compare *DvPdfG4/Clk*Δ; *tubG80/*+, *DvPdfG4/*+; *tubG80/*+, and *Clk*Δ/+; *tubG80*/+). CLKΔ expression in adult LNvs reduces the levels and diurnal rhythms of PDF accumulation in the s-LNv dorsal projections (Figures 2B,C) as is the case with adult-restricted PER RNAi. These results validate that adult-restricted expression of CLKΔ using GAL4/GAL80ts is immediately in effect and further suggest that loss of PER rhythms and reduction in axonal PDF levels in the s-LNvs during adulthood do not necessarily impair morning anticipation. These data instead suggest the involvement of other factors that are regulated by CLK in generating morning anticipation.

In this regard, it is noteworthy that morning anticipation was reduced in the flies expressing TNT in adult LNvs compared with controls and even further reduced when CLKΔ and TNT were co-expressed (Figures 7A,B, compare *TNT/*+; *gal1118/tubG80*, *CLK*Δ*/TNT; gal1118/tubG80, TNT/*+; *tubG80/*+, and *gal1118/tubG80*). As TNT is known to block both neurotransmitter and neuropeptide release (Sweeney et al., 1995; Ding et al., 2019), the results suggest the possibility that, in addition to the PDF, neurotransmitter, such as glycine (Frenkel et al., 2017) or the short Neuropeptide F (sNPF) (Johard et al., 2009), may be involved in the normal morning anticipation.

We also noticed that evening activity peak was not apparently advanced in all the genotypes tested (Figure 7A). Free-running rhythms were not shortened either (Table 1). This was surprising because loss of PDF advances evening peak in a 12 h:12 h LD cycle at the temperature near 25°C (Renn et al., 1999). The lack of effect on evening activity even in the flies that have reduced morning anticipation is probably because their PDF levels are only partially reduced (Figures 2B,C, 3B,C). Additionally, the fact that all the behavioral experiments were performed at 29°C, the temperature that suppresses daytime activity (Majercak et al., 1999; Parisky et al., 2016), likely masked the effects on the evening activity peak.

## Discussion

In this study, we disrupted molecular clocks or neural transmission only in adulthood in restricted subgroups of pacemaker neurons to better understand the network property of the circadian circuit. Our results are summarized in three main points: (i) free-running locomotor rhythms are maintained for several days while molecular clocks are disrupted in the M-oscillator or in both M- and part of E-oscillators; (ii) morning anticipation does not require PER cycling but requires intact CLK; and (iii) disruption of M-oscillator’s neuronal output dampens free-running rhythms, and the disruption of both clocks and neural output of the M-cells results in an immediate behavioral arrhythmia under constant conditions. These results are largely in line with previous findings, with minor deviations.

Recent studies used cell-specific CRISPR knockout of *per* or *tim* and showed that the absence of clocks in the M-cells does not impair locomotor rhythms (Delventhal et al., 2019; Schlichting et al., 2019). Adult-specific expression of CLKΔ or PER RNAi in the M-cells recapitulates their findings. Whereas CRISPR-mediated clock knockout in both the M-cells and LN-EO causes an immediate behavioral arrhythmia (Delventhal et al., 2019; Schlichting et al., 2019), adult-restricted expression of CLKΔ with *DvPdf-GAL4*, which is expressed in the M-cells and two out of four LN-EO cells, gradually dampens the locomotor rhythmicity over several days. This difference is probably due to the fact that clocks in the two CRY- and sNPF-positive LNds are not disrupted in *DvPdf* > *CLK*Δ flies and can contribute to maintaining rhythmic locomotor output. Therefore, CRY-positive LNds, which play a crucial role in driving evening activity in LD, might also promote free-running rhythms in DD (Rieger et al., 2009). Additionally, the onsets of clock disruption differ between our study and clock knockout studies; the exact onset of gene deletion may not be reliably determined when using the GAL4-driven CRISPR knockout strategy, especially because many GAL4s are also expressed during development. Despite subtle differences, these two results are not mutually contradictory and both support that clocks in the M-oscillator are dispensable during adulthood for maintaining free-running locomotor rhythms as long as clocks in other pacemakers, including CRY-positive LN-EO, are intact (Figure 7C).

Adult-restricted electrical silencing of the M-cells does not affect M-cells’ clocks but leads to a gradual dampening of behavioral rhythms and eventual arrhythmia (Depetris-Chauvin et al., 2011). We show that TNT expression in adult M-cells recapitulates this phenotype. Importantly, adult-restricted expression of both TNT and CLKΔ in the M-cells results in an immediate behavioral arrhythmia. Therefore, lack of molecular clockwork is compensated as long as the M-oscillator can produce synaptic and/or peptidergic output. Conversely, lack of neural transmission from the M-oscillator can be overcome as long as their internal clocks are functional.

How can the circadian circuit maintain rhythmic locomotor output while the clocks or neural function is disabled in the M-cells? It has been shown that TNT expression in the *DvPdf-GAL4* positive, PDF-negative cells (i.e., the 5th s-LNv, three CRY negative LNds and one CRY-positive, ITP-positive LNd) alone during adulthood renders flies arrhythmic (Guo et al., 2014). Constitutive electrical silencing of the *DvPdf-GAL4* positive, PDF-negative cells severely disrupts locomotor rhythms, whereas silencing of three CRY-positive LNds and the 5th s-LNv labeled by the MB-122b split GAL4 only during adulthood reduces rhythm power without affecting clocks in the M-cells (Bulthuis et al., 2019). The LN-EO cells make synaptic contacts onto the M-cells and rhythmically modulate their excitably (Duhart et al., 2020). Genetic rescue of *per*^0^ flies with the *Clk4.1M* driver restores morning activity but is unable to rescue arrhythmic DD behavior (Zhang Y. et al., 2010). Collectively, these findings suggest that the LN-EO can input signals to the M-cells, through which behavioral output is maintained even when the M-cells are clockless. Intriguingly, changing the pace of the LN-EO does not alter period or power of locomotor rhythms in DD when clocks in the M-cells are intact (Chatterjee et al., 2018). Therefore, role switching from the M-cells to LN-EO seems to occur only when clocks in the M-cells are dysfunctional. DN1p, on the other hand, is strongly coupled to the M-cells and are the major output route of the M-cells (Chatterjee et al., 2018). When M-cells are clockless, the LN-EO or other pacemaker neurons excluding DN1ps, might also output locomotor rhythms without passing through the M-cells, since the electrical silencing and TNT expression of the M-cells alone does not immediately disrupt locomotor rhythms. There is also a possibility that M-cells’ clocks may control locomotor output in a manner resistant to TNT, such as via gap junctions (Schneider and Stengl, 2006; Ramakrishnan and Sheeba, 2020) or via TNT-insensitive transmitters/peptides.

Previous works have shown that the M-cells can function as the cell-autonomous driver of the morning anticipation when harboring functional clocks but its function can be modulated by the DN1p or other pacemakers (Stoleru et al., 2004; Cusumano et al., 2009; Zhang Y. et al., 2010; Menegazzi et al., 2020). Our results of the adult-restricted PER knockdown in the M-cells are congruent with these conclusions. However, CLKΔ expression in adult M-cells significantly reduces the morning anticipation, suggesting that intact CLK within the M-cells is required for morning anticipation behavior. Both PER knockdown and CLKΔ expression reduce PDF levels and rhythms in the dorsal termini of the M-cells; therefore, factors other than PDF are involved in controlling the morning anticipation. The report that M-cell-specific ablation of *vrille* reduces PDF expression and rhythms via post-transcriptional regulations but does not affect morning anticipation (Gunawardhana and Hardin, 2017) also supports this interpretation. Taken together with our finding that expression of TNT in adult M-cells reduces the morning anticipation, these results suggest that a certain neurotransmitter or sNPF is under the control of CLK and plays an important role in driving morning anticipation behavior. Additionally, it is noteworthy that both CLKΔ expression and *per* knockdown in the M-cells reduce PDF levels and rhythms in the dorsal termini of the M-cells but does not immediately deteriorate locomotor rhythmicity. These results confirm the previous report that PDF rhythms in the dorsal projections do not play important roles in locomotor rhythms in DD (Fernandez et al., 2020).

In summary, the present study highlights the remarkable resilience of *Drosophila* circadian pacemaker circuit, the property conserved in mammals (El Cheikh Hussein et al., 2019). Our findings support the emerging view that the topology of the pacemaker circuit is not rigid, as in the classical M- and E-oscillator model, but rather flexible, assigning different neuronal subgroups the task of pacemaking in order to achieve the resilience.

## Data Availability Statement

The original contributions presented in the study are included in the article/Supplementary Material, further inquiries can be directed to the corresponding author/s.

## Author Contributions

EJ and EN: conceptualization. EJ, RK, and EN: investigation. EN: writing and funding acquisition. All authors contributed to the article and approved the submitted version.

## Conflict of Interest

The authors declare that the research was conducted in the absence of any commercial or financial relationships that could be construed as a potential conflict of interest.
