# Supplementary material for: Uncovering the Roles of Clocks and Neural Transmission in the Resilience of Drosophila Circadian Network
Source: Front Physiol. 2021 May 26;12:663339. doi: 10.3389/fphys.2021.663339 (PMC8188733; doi:10.3389/fphys.2021.663339)
Supplement: Supplementary file 1 [file Data_Sheet_1.DOCX]

Supplementary Material


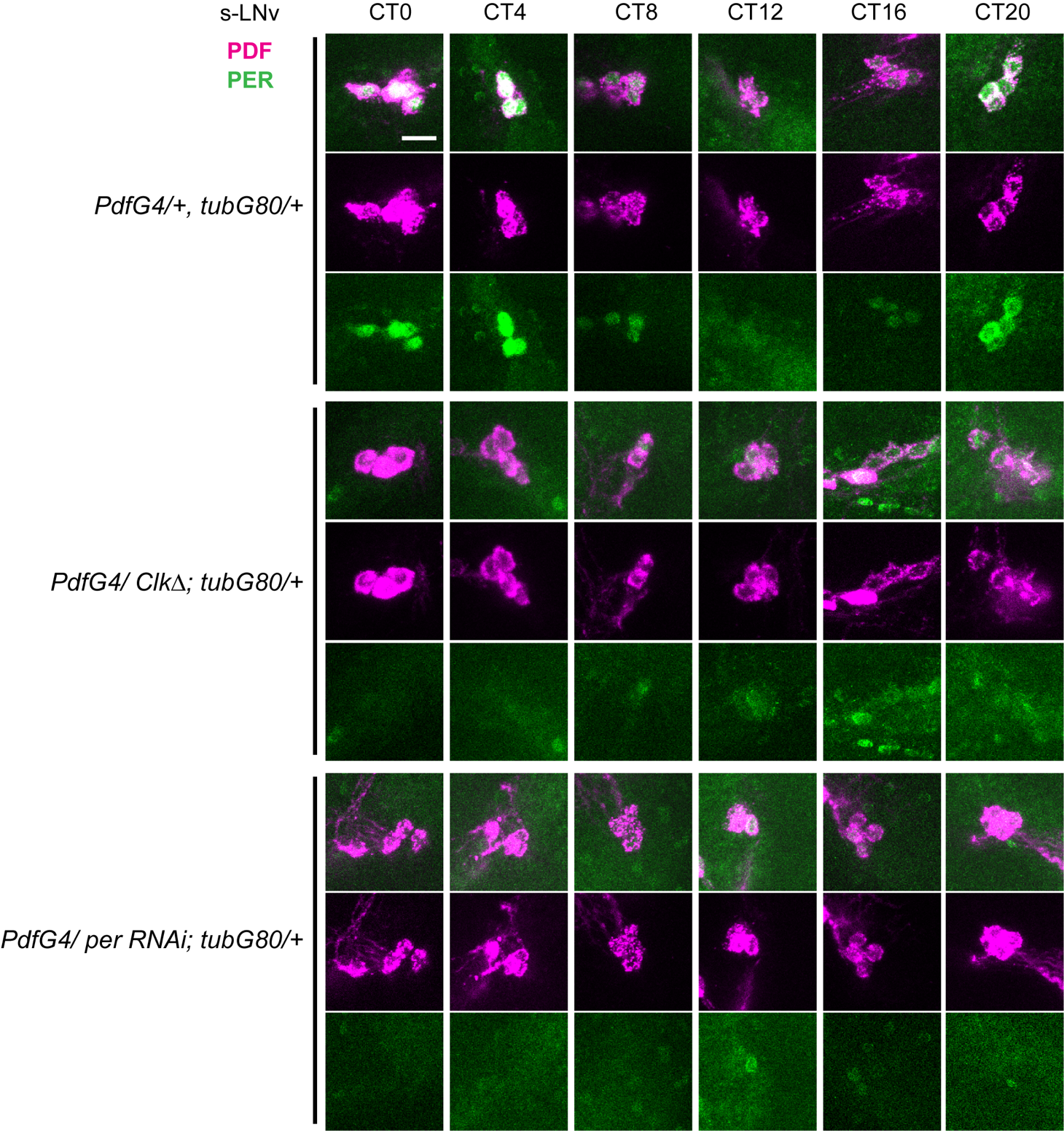


**Supplementary Figure S1.** Representative confocal images of the s-LNvs immunostained for PER (green) and PDF (magenta) on DD3 during the adult-restricted GAL4 activation. Top, driver-only control (*PdfG4/+; tubG80/+* stands for *Pdf-GAL4/+; tub-GAL80ts/+*). Middle, s-LNvs expressing *ClkΔ* in adulthood (*PdfG4/Clk∆; tubG80/+* stands for *Pdf-GAL4/UAS-Clk∆; tub-GAL80^ts^/+*). Bottom, s-LNvs expressing *per* RNAi in adulthood (pdfG4/ perRNAi; tubG80/+ stands for *Pdf-GAL4/UAS-per RNAi; tub-GAL80^ts^/+*). Scale bar, 10 μm.


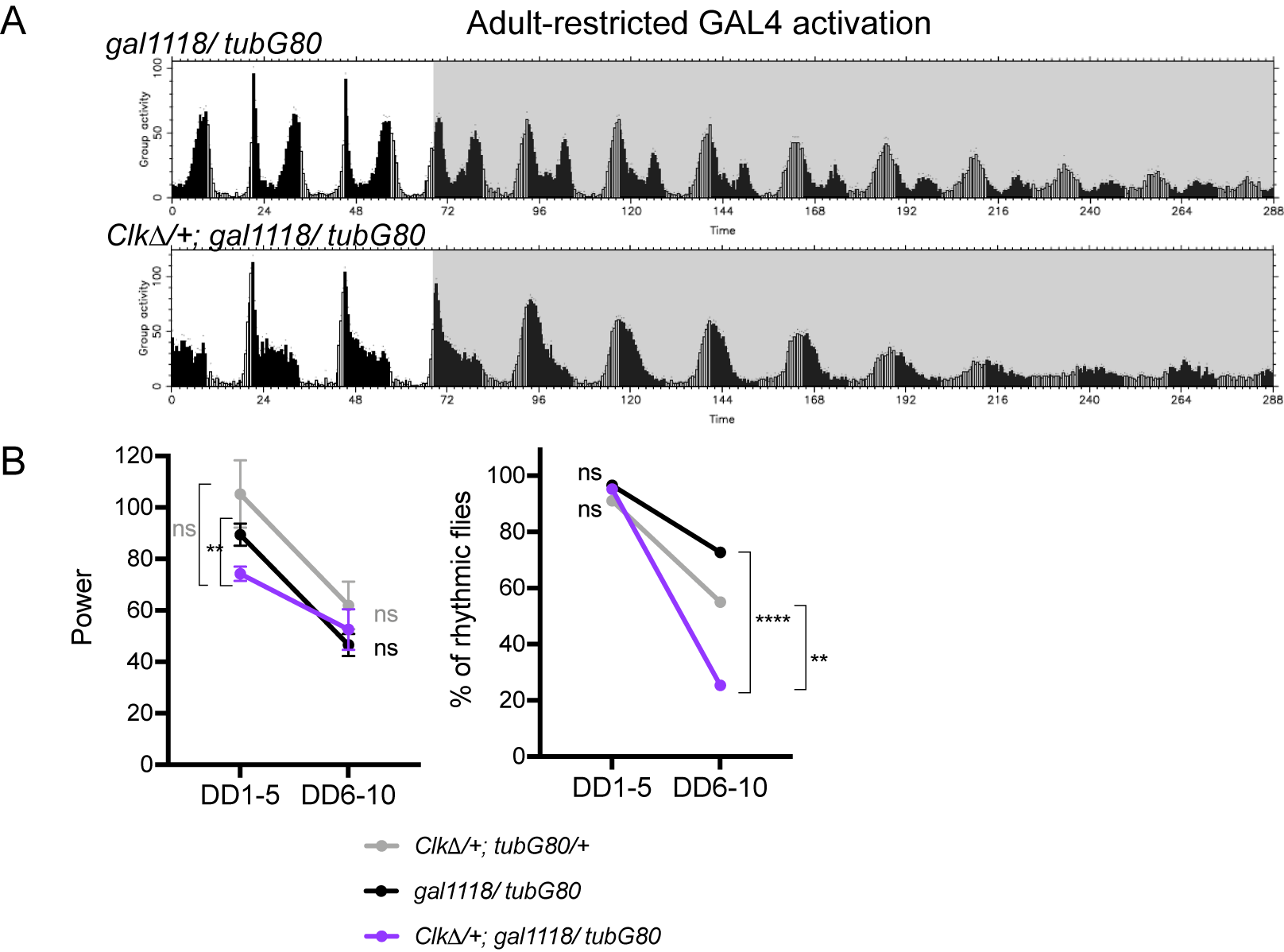


**Supplementary Figure S2.** Effects of conditional expression of CLKΔ on locomotor rhythms. *UAS-ClkΔ* was expressed in the LNvs only during adulthood with the combination of *gal1118* and *tub-GAL80^ts^* and a temperature shift from 18℃ to 29℃. (A) Group average locomotor activity. (B) Left, power of rhythmicity in the flies of indicated genotypes in DD1-5 and DD6-10. **p<0.01 by the unpaired t-test with Welch’s correction. Right, percentage of the rhythmic flies in DD1-5 and DD6-10. **p<0.01 and ****p<0.0001 by Fisher’s exact test. ns, not significant.
